# A Compression-Based Method for Detecting Anomalies in Textual Data

**DOI:** 10.3390/e23050618

**Published:** 2021-05-16

**Authors:** Gonzalo de la Torre-Abaitua, Luis Fernando Lago-Fernández, David Arroyo

**Affiliations:** 1 Departamento de Ingeniería Informática, Escuela Politécnica Superior, Universidad Autónoma de Madrid, 28049 Madrid, Spain; gonzalo.torreabaitua@gmail.com (G.d.l.T.-A.); luis.lago@uam.es (L.F.L.-F.); 2 Institute of Physical and Information Technologies (ITEFI), Spanish National Research Council (CSIC), 28006 Madrid, Spain

**Keywords:** intrusion detection systems, anomaly detection, normalized compression distance, text mining, data-driven security

## Abstract

Nowadays, information and communications technology systems are fundamental assets of our social and economical model, and thus they should be properly protected against the malicious activity of cybercriminals. Defence mechanisms are generally articulated around tools that trace and store information in several ways, the simplest one being the generation of plain text files coined as security logs. Such log files are usually inspected, in a semi-automatic way, by security analysts to detect events that may affect system integrity, confidentiality and availability. On this basis, we propose a parameter-free method to detect security incidents from structured text regardless its nature. We use the Normalized Compression Distance to obtain a set of features that can be used by a Support Vector Machine to classify events from a heterogeneous cybersecurity environment. In particular, we explore and validate the application of our method in four different cybersecurity domains: HTTP anomaly identification, spam detection, Domain Generation Algorithms tracking and sentiment analysis. The results obtained show the validity and flexibility of our approach in different security scenarios with a low configuration burden.

## 1. Introduction

We live in a complex world with multiple and intricate interactions among countries, companies and people. Those relations are preferentially conducted by Information and Communication Technologies (ICT) [1]. As the number of devices that are connected to the Internet has increased, so has grown the number of malicious agents that try to get profit from systems vulnerabilities. These malicious actors can target governments, companies or individuals using several kinds of attacks. Malicious activities range from simple attacks such as parameter tampering, spam or phishing, to more complex menaces such as botnets, Advanced Persistent Threats (APTs) or social engineering attacks that leverage Domain Generation Algorithms (DGAs) and Open Source Intelligence techniques (OSINT) [2,3]. In order to help to protect their networks, systems and information assets, cybersecurity analysts use different tools, such as Intrusion Detection Systems (IDS), Firewalls or Antivirus. These tools generate huge amounts of information in the form of text logs with a blend of normal behaviors and malicious interactions. Moreover, the proper implementation of the security life-cycle is highly dependent on the adequate aggregation of additional records of activity and other sources of digital evidence [4,5]. Due to the ever increasing size of security related data, the automatic treatment and analysis of text logs is a key component in the deployment of adaptive and robust defensive strategies [6]. Therefore, it is interesting to have a single methodology that can be used with these logs to simplify and help analysts with their duties and decision making. Our approach consists of developing parameter-free methods for such a goal [7].

As a departing point, we are considering five binary classification problems in four different cybersecurity related areas that enclose anomalous versus normal URLs, spam versus non spam messages, normal versus malicious Internet domains and malicious versus normal messages in fora or social networks. This is a heterogeneous landscape where the common nexus is determined by sources of information that can be treated as text. It can be shown that the use of compression information techniques comes helpful to process textual data with different codification systems. Indeed, these techniques have been successfully used in several domains, such as clustering and classification of genomic information [8], language identification [9], plagiarism detection [10] or URL anomaly detection [11].

In this work, we explore and validate the use of a previous method based on compression information techniques [12] in a wider scenario that encompasses several binary classification problems in the current cybersecurity context. These include spam detection, malicious URL identification, DGA targeting and sentiment analysis in Twitter and in movie reviews. We use the Normalized Compression Distance (NCD) [10] to extract a set of features that characterise the textual data associated to each problem, and train a Support Vector Machine (SVM) that classifies the data using these features. Our results on the different problems show the generality of this approach, which shows up as a unified framework for the analysis of textual information in a general cybersecurity context. Indeed, although we do not improve the best state of the art solutions in each considered problem, we obtain competitive results in all the domains. The slight accuracy decrease is off-set by the surplus represented by the versatility of the methodology.

The rest of the paper is organized as follows: Section 2 describes previous works on each of the considered domains, Section 3 describes the proposed method, Section 4 explains the details and results of each of the experiments and Section 5 outlines the conclusions.

## 2. Anomaly Detection in Cybersecurity

This section discusses different strategies followed by the cybersecurity community to tackle some of the threats they face. Traditional approaches involve the use of anomaly detection techniques, which have been used, for example, for virus or intrusion detection. More recently, with the rise of social networks and open sources of information, the attack surface has increased [2]. To overcome these new risks, cybersecurity researchers have started using Natural Language Processing (NLP) techniques, such as sentiment analysis. For instance, NLP has been applied to detect cyberbulling [13], malware [14], or to predict cyber-attacks [15]. In this article we focus on four representative domains where the analysis of textual information is also relevant. In the following paragraphs we describe the problems and the main approaches used to cope with them.

### 2.1. HTTP Anomaly Detection

Usually, the detection of anomalies in the cybersecurity field is done using IDS [16]. IDS can be targeted at analysing either network or host activity. Moreover, we can adopt a static approach by comparing activity traces to concrete patterns or signatures, or we can apply a dynamic approach in terms of behaviour analysis. The latter is the one that has focused most research efforts. The techniques for HTTP traffic anomaly detection can be classified into seven general groups: statistical models, static analysis, statistical distances, data mining and pattern analysis, Markov models, machine learning and knowledge based [16,17,18,19,20,21]. Different features of the HTTP packets and the HTTP protocol, including the text of the URL, are used to model the normal network behaviour.

### 2.2. Spam Detection

Spam detection is another area that has been broadly studied in the cybersecurity context. Several machine learning techniques have been applied, ranging from Naïve Bayes to logistic regression or neural networks [22]. As this problem has a big dependence on text, text analysis and clustering techniques have also been widely studied, with the Term Frequency (TF) and Term Frequency Inverse Document Frequency (TFIDF) statistics [23] usually applied for attribute construction, as well as edit distances [24]. Of particular interest to our work is the application of compression based techniques [25,26]. These have a good performance and are quite robust against noise in the channel that eventually could induce an erroneous classification [27].

### 2.3. DGA Detection

DGA is a technique used by malware to conceal its Command and Control (C&C) panel. It consists of the generation of random domain names and is one of the techniques used by botnets (groups of hijacked computers and infected devices (https://www.trendmicro.com/vinfo/us/security/definition/botnet, last access 16 February 2021)) to communicate and hide the real address of its C&C.

The main techniques applied to detect DGA domains analyse the Domain Name System (DNS) traffic in a specific network. They use features such as the frequency and the number of domains that do not resolve (NXDomain) to cluster and identify malware families [28,29]. Another approach involves the use of the domain name to identify whether it has been generated by a DGA. This is done by the detection of patterns, and usually requires a feature extraction step before applying algorithms such as Neural Networks [30,31] or n-gram categorisation [32,33].

### 2.4. Sentiment Analysis

Sentiment analysis is a field of text mining that has attracted the attention of cybersecurity researchers. Some examples of application of sentiment analysis in the security context include the identification of threats [34], radicalism and conflicts in fora [35], the detection of cybercrime facilitators in underground economy based on customer feedback [36], the characterisation of disinformation phenomena [37,38], and the use of Twitter to anticipate attacks [39] and generate alerts [40]. The main strategies consist of a feature selection step using techniques such as TFIDF or Point-wise Mutual Information (PMI), followed by a sentiment classification step using either machine learning or lexicon based approaches [41,42]. Deep Learning techniques, including Convolutional Neural Networks [43,44] and Word Embeddings [45], are also considered in this context.

## 3. Materials and Methods

It is worth noting that most of the previously reviewed techniques are problem specific. Even though all the presented problems involve some kind of analysis of textual information, the field lacks a general methodology that faces all these issues from a common perspective. This section describes a general mechanism to extract a set of numerical attributes that characterize a text using a compression based metric, such as the NCD. Given a text *T*, the main idea is to compute the distance between *T* and a set of *k* additional texts $\left\{g_{1},g_{2},\ldots ,g_{k}\right\}$, known as *attribute generators* (AGs). This provides a vector of *k* numbers that represents the text *T* in a *k*-dimensional attribute space, and can be used as input for a classification algorithm [12]. Although in this work we have used a SVM classifier, it is necessary to highlight that our methodology for attributes extraction can be used with any other classification algorithm.

For a dataset consisting of text strings belonging to two different classes, the detailed procedure is as follows. We randomly divide the data into two disjoint groups, *G* and *I*, with *m* and *n* texts, respectively. Both groups are balanced, meaning that they contain the same number of strings for each of the two classes. The first group, *G*, is used to build the attribute generators. To do so, the strings in *G* are packed into a set of *k* generator files, each one with strings from one class only. Thus we have k/2 generators for each class. The second group, *I*, contains the set of *n* strings that are used to train the classifier, after being characterized by the distances to the attribute generators. That is, for each string $s_{i}$ in *I*, we compute the distance between $s_{i}$ and each of the *k* attribute generators to obtain the vector:

(1) $$S_{i}=\left(x_{i1},x_{i2},\ldots ,x_{ik}\right)$$

which is used to characterize the string $s_{i}$. The components $x_{ij}$ of $S_{i}$ are given by:

(2) $$x_{ij}=D\left(g_{j},s_{i}\right)$$

where $D(g_{j},s_{i})$ is the normalized conditional compressed information [10] between the generator $g_{j}$ and the string $s_{i}$:

(3) $$D\left(g_{j},s_{i}\right)=\frac{C(s_{i}|g_{j})}{C(s_{i})}=\frac{C\left(g_{j}\cdot s_{i}\right)-C\left(g_{j}\right)}{C(s_{i})}$$

C(x) is the compressed size of *x* and the dot operator represents concatenation. In the experiments we use the *gzip* compressor because it has a better speed performance than other compression algorithms [46].

**Figure 1:**
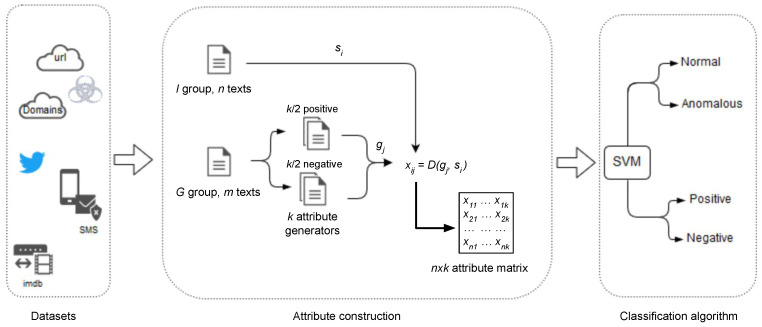
End to end representation of the proposed method. The set of texts composing the dataset are first divided into two groups, *G* and *I*. The *m* texts in the *G* group are further divided into *k* additional groups, or *attribute generators*. Then the distance between each text in *I*, $s_{i}$, and each generator $g_{j}$ is computed in order to obtain a n×k attribute matrix which is used to train a SVM.

Once the attribute vectors have been generated for all the strings in *I*, we use them to train a SVM with a radial basis function (RBF) kernel that predicts the class associated to each string. We use the *scikit-learn* Python library [47] for the implementation, and measure the quality of the classifiers by using the accuracy (ACC) and the area under the receiver operating characteristic curve (AUC) metrics. The SVM model depends on two hyperparameters, the complexity *C* and the kernel width γ, which are tuned, for each *k*, using a standard 5-fold cross-validation strategy as described in [12]. The results presented in Section 4 are averaged over 10 different partitions of the data into the *G* and *I* sets. Figure 1 shows the end to end process, from the initial dataset to the final classification.

## 4. Results

To assess the validity of the proposed method, we have performed experiments in four different and heterogeneous domains. Concretely, we have explored the detection of malicious HTTP requests (Section 4.1), the identification of spam in SMS messages (Section 4.2), the detection of DGA domains (Section 4.3) and the analysis of sentiment both in Twitter (Section 4.4) and in movie reviews (Section 4.5). All the problems consist of a set of strings belonging to two different classes, for example normal versus anomalous HTTP requests or positive versus negative sentiment in tweets. Nevertheless the characteristics of each dataset are unique, and we observe a high variability both with respect to the string length (Table 1) and the string content (Tables 2, 4, 6, 8 and 10). Despite this variability, the features extracted following the presented method are able to provide a good description of the problem data in all the cases, and the classifiers trained on them obtain state of the art accuracy. The following subsections describe in detail each of the experiments carried out.

### 4.1. Experiment 1—Malicious URL

This experiment tackles the issue of identifying a malicious HTTP request only using the related URL string. Usually, this problem has been faced analysing additional information, such as the URL length, the number of URL parameters or the parameter values [48]. Our method simplifies the preprocessing step by considering the raw text of the URL, hence avoiding any sort of manual attribute construction.

#### 4.1.1. Data Preparation

We use the public CSIC-*2010* dataset [49], which contains examples of both normal and anomalous HTTP requests. We extract all the POST requests, remove duplicates and balance the queries. This results in a total of 9600 queries, 4800 of each class. After this preprocessing step, we divide the dataset into the *I* set, with 1600 randomly chosen queries (800 normal and 800 anomalous) and the *G* set, with 8000 queries (4000 normal and 4000 anomalous). Some examples of normal and anomalous queries are shown in Table 2.

#### 4.1.2. Results

We have performed experiments using a number of attributes, *k*, ranging from 8 to 160. The results can be seen in Table 3. The highest accuracy is obtained for k=80, with a 95% of correctly classified HTTP queries. This value is similar to other results reported in the literature [50]. Nevertheless, our approach does not require a feature selection step and depends on a smaller number of hyperparameters.

### 4.2. Experiment 2—Spam

In this experiment we apply the method to the problem of discriminating between legitimate and spam SMS messages. One of the main characteristics of this kind of messages is that they are usually written using a very informal language, with many invented terms and abbreviations which are not always grammatically correct. This fact may be a problem for traditional NLP methods based on lemmatisation or parsing [51]. The method here proposed is however agnostic to the grammar or the rules followed by the messages, and it can be directly applied to this problem without any adaptation. The next paragraphs describe the data preparation and the results obtained.

#### 4.2.1. Data Preparation

We use the SMS Spam Collection v.1, a public dataset that can be obtained from the authors’ page (http://www.dt.fee.unicamp.br/~tiago/smsspamcollection, last access 16 February 2021) and also from Kaggle (https://www.kaggle.com/uciml/sms-spam-collection-dataset/data, last access 16 February 2021). It contains 5574 SMS messages, 747 of them labeled as *spam* and the rest, 4827, labeled as neutral, or *ham*. We balance the dataset by taking all the spam messages and randomly selecting a sample of 747 ham messages. The balanced data are further divided into the *I* set, with 400 messages (200 ham and 200 spam), and the *G* set, with 1094 messages (547 of each class). Table 4 shows some examples of ham and spam messages.

#### 4.2.2. Results

As before, we have performed experiments for *k* ranging between 8 and 160. A summary of the results can be found in Table 5. We observe an increase of performance as more attribute generators are used, with a maximum of 0.96 AUC and 0.904 accuracy for k=160. These values are slightly worse than the best results reported in the literature for the same dataset [52], but the latter need a more complex and problem specific preprocessing step which is avoided if using the proposed method, with the subsequent simplification of the overall process.

### 4.3. Experiment 3—DGAs

In the third experiment we apply the method to the detection of DGAs relying on the domain name only. The main characteristic of this problem, which makes an important difference with respect to the rest of considered scenarios, is that the string lengths are significantly shorter. It is very unlikely that a domain name contains more than 100 characters (see Table 1). In spite of this fact the proposed approach has been applied with no adaptations, and the results are quite satisfactory.

#### 4.3.1. Data Preparation

We use a dataset where the legitimate domain names are extracted from the Alexa top one million list (https://www.alexa.com/topsites, last access 16 February 2021), whilst the malicious domains are generated with 11 different malware families, such as zeus, cryptolocker, pushdo or conficker. The dataset can be downloaded from Andrey Abakumov’s GitHub repository (https://github.com/andrewaeva/DGA, last access 16 February 2021). Raw data contain 1,000,000 normal and 800,000 malicious DGA domains. After balancing the classes, we randomly select a subset of 13,000 domains, 6500 for each class, and from them we use 800 domains as the *I* set. The remaining 5700 domains are used to build the attribute generators (*G* set). Table 6 shows some examples of both DGA and normal domain names.

#### 4.3.2. Results

A summary of our results on the DGA dataset can be seen in Table 7. As in previous experiments, we have carried out tests with different *k* values. The classification accuracy increases with *k* up to a point where it saturates. The best results are obtained for *k* = 80, with an accuracy of 0.94 and an AUC of 0.98. These values are better than those reported when using traditional methods, although they can be improved by using deep neural models such as recurrent neural networks [53]. Note however that we are using only a small subset of the original data to train the classifier.

### 4.4. Experiment 4—Sentiment Analysis in Twitter

For this experiment we use the *Sentiment140* dataset (http://help.sentiment140.com, last access 16 February 2021.) described in [54], which contains a training set with 1,600,000 tweets labeled as either positive or negative according to their sentiment. There are 800,000 positive and 800,000 negative tweets, collected between 6 April and 25 June 2009, and labeled using the emoticons contained within the message. Table 8 shows a sample of 5 positive and 5 negative tweets extracted from the training set. The dataset also contains a small test set with 498 tweets which were labeled manually, to be used for validation purposes.

#### 4.4.1. Data Preparation

In our experiments we use only the tweets in the training set. Prior to our analysis we preprocess the data in order to remove both duplicated tweets and tweets that appear both as positive and negative. After this preprocessing stage we obtain a new training set with 787,956 tweets of each class. The whole text of the messages, without any further preprocessing, is used to characterize the tweets. The *I* and *G* sets are built as in previous sections. In particular we use 50,000 tweets of each class to build the attribute generators. The remaining messages are used to train the classifiers after being characterized by their distance to the generators. In this case, due to the dataset size, we use a SVM classifier with a linear kernel.

#### 4.4.2. Results

The results are shown in Table 9. The accuracies in the table are slightly worse than those reported in [54], but there are two important points to consider. First, they perform additional preprocessing of the tweets. In particular, they replaced all usernames by the new token *USERNAME*, they replaced all URLs by the keyword *URL*, and they eliminated repeated letters in order correct some uses of informal language usually present in tweets. In this article we have decided to omit these steps in order to show the generality of our approach. Second, their results are obtained on the test set, which contains only 498 tweets. Results on such a small dataset may be biased. In fact, when we evaluate our method on these data, we observe a systematic increase of both the accuracy and the AUC.

### 4.5. Experiment 5—Sentiment Analysis in Movie Reviews

In this last scenario we tackle a sentiment analysis problem in movie reviews. It has the particularity that the strings are of arbitrary length. Concretely, the average length of the movie reviews in the dataset is 1325 characters for the positive reviews and 1294 for the negative reviews (see Table 1). This characteristic contrasts with the Twitter problem, where the string length is limited to 140 characters. Besides, the use of language and grammar tends to be more formal, and the inclusion of abbreviations and emoticons is not so extended. These are fundamental differences that motivate the application of the proposed method in this problem.

#### 4.5.1. Data Preparation

We use a public dataset from the Stanford NLP group (http://ai.stanford.edu/~amaas/data/sentiment/, last access 16 February 2021). It contains 50,000 movie reviews extracted from the Internet Movie Database (IMDb) (https://www.imdb.com/, last access 16 February 2021). Each review is a text string commenting a movie, and classified as either positive or negative. There are 25,000 reviews classified as positive and 25,000 classified as negative. Table 10 contains samples of both classes. From these raw data we use a subset of 5350 randomly chosen reviews (half positive, half negative). The *I* and *G* string groups are build as for previous problems. The *I* set contains 1600 movie reviews (800 positive and 800 negative), and the *G* set contains the remaining 3750 texts.

#### 4.5.2. Results

This experiment has been carried out using different *k* values as in previous sections. In this case the best results are obtained for k=160 (acc.=0.86, AUC=0.93, see Table 11). Although these results do not improve the best reported for the same dataset [55], they are quite competivite, even more if we take into account that they have been obtained on a small subsample of the original data.

## 5. Conclusions

Information security calls for a comprehensive deployment of protection measures and detection controls [56]. Security logs are the core of such controls, since they enable event recording and attacks characterization. Anomaly detection in security logs is one of the most relevant means to detect possible malicious activity. However, those security logs are derived from plenty of different network and information flow modalities. Therefore, there exists an urge to adopt mechanisms to process security information regardless of the concrete nature of each log [57]. In this vein, we have proposed a method that is able to process textual data from different sources using a common approach: the NCD is used to extract features that characterize the text, and a SVM is trained on these features to perform the classification.

To test the method, we have performed five experiments over four different domains. Our results are competitive in general, although for some problems we obtain a classification accuracy slightly below the best values reported in the literature. Nevertheless, it is worth noting that we are using a unique procedure to address all the problems despite their disparate nature. In other words, we have sacrificed accuracy for adaptability. In addition, we are using much less data to train the classifiers than other proposals in the literature, since part of the available data are used to build the attribute generators. The use of all training data, following the approach in [58], could further improve the results here presented. Moreover, additional efforts are required to test the suitability of our methodology in multiclass classification problems in cybersecurity (as those in [59,60]).

Another advantage of our method is that it neither needs to perform a preprocessing step nor to manually construct features from the data, and the hyper-parameter tuning is minimal. The number of generated attributes, *k*, appears to be the most relevant parameter. In general, in all the problems under consideration we observed an increase in performance as *k* grows, saturating for *k* large enough. We have found that the optimal *k* values are related to the sliding window of the gzip compressor. When *k* is small, the size of the generator files is usually larger than this sliding window, and hence not all the information contained in the attribute generator files is used to characterize the string instances. On the other hand, increasing the number of generators by reducing their size below the sliding window size does not seem to further improve the classification accuracy. The use of other compressors has not been considered in the present study. Future work should be devoted to test how different compression algorithms may affect our results.

All in all, our proposal leads to an adequate trade-off between adaptability and performance, and it can be interpreted as a complementary procedure in frameworks tackling the limitations of the “no free lunch” theorem [61] by the convenient integration of several anomaly detection methods. This convenient integration demands an exhaustive study of how to improve the method by using different compression algorithms to calculate the NCD and estimate Kolmogorov complexity. Besides, it would be advisable to analyze the impact of using other models than the SVM to conduct classification in the *k* dimensional hyperspace determined by our NCD-based methodology.

## Figures and Tables

**Table 1:** Average (μ), standard devivation (σ), and quartiles Q1, Q2 and Q3 of the string length for each of the problems and classes.

	URL		DGA		Spam		Twitter		Movie Reviews	
	Normal	anom.	Normal	DGA	Normal	Spam	posit.	negat.	posit.	negat.
μ	138	147	15	20	72	139	74	74	1325	1294
σ	97	107	5	7	58	29	36	37	1032	946
Q1	59	63	11	15	33	133	44	44	691	706
Q2	71	85	14	20	52	149	69	70	968	973
Q3	248	251	17	28	93	157	103	104	1614	1568

**Table 2:** Sample strings of 4 normal and 4 anomalous queries in the *CSIC-2010* dataset.

Four Examples of Normal Queries
1. modo=registro&login=beveridg&password=camale%F3nica&nombre=Stefani&apellidos=Gimeno+Cadaveira&
email=morando%40sandrasummer.gy&dni=91059337Z&direccion=C%2F+Bobala+111+4A&ciudad=Mog%E1n&
cp=46293&provincia=Castell%F3n&ntc=8975566519527853&B1=Registrar
2. id=1&nombre=Queso+Manchego&precio=39&cantidad=57&B1=A%F1adir+al+carrito
3. modo=insertar&precio=5588&B1=Confirmar
4. modo=registro&login=ouellett2&password=radicalmente&nombre=Ranquel&apellidos=Orra&
email=hodo%40deltamarina.my&dni=18518539C&direccion=Fructuoso+Alvarez%2C+55+&
ciudad=Bay%E1rcal&cp=17742&provincia=Palencia&ntc=3322562441567993&B1=Registrar
**Four Examples of Anomalous Queries**
1. modo=insertar&precioA=900&B1=Pasar+por+caja
2. modo=entrar&login=dedie&pwd=M50879RIST44&
remember=bob%40%3CSCRipt%3Ealert%28Paros%29%3C%2FscrIPT%3E.parosproxy.org&B1=Entrar
3. modo=registro&login=alix&password=venI%21A&nombreA=Imelda&apellidos=Delb%F3n+Coll&
email=holister%40brunoseguridad.cf&dni=80525673M&direccion=Plza.+Noria+De+La+Huerta+68%2C+&
ciudad=Alcudia+de+Veo&cp=28690&provincia=%C1vila&ntc=6551003767368321&B1=Registrar
4. modo=entrar&login=bienek&pwd=cloqu%27e%2Fro&remember=off&B1=Entrar

**Table 3:** Results on the HTTP problem. Mean accuracy (Acc.) and area under ROC (AUC) for each of the considered *k* values. The best values are shown in boldface.

*k*	*C*	γ	Acc.	AUC
8	1.0	100.0	0.84±0.03	0.909
16	10.0	10.0	0.88±0.03	0.946
32	1.0	10.0	0.93±0.02	0.968
80	1.0	10.0	**0.95** ± **0.02**	**0.975**
160	2.0	5.0	0.95 ± 0.02	0.974

**Table 4:** Sample strings of several ham and spam messages in the SMS Spam Collection v.1 dataset.

Four Examples of Ham SMS
1. What you doing?how are you?
2. Ok lar... Joking wif u oni...
3. Cos i was out shopping wif darren jus now n i called them 2 ask wat present he wan lor. Then he
started guessing who i was wif n he finally guessed darren lor.
4. MY NO. IN LUTON 0125698789 RING ME IF UR AROUND! H*
dun say so early hor... U c already then say...
**Four Examples of Spam SMS**
1. FreeMsg: Txt: CALL to No: 86888 & claim your reward of 3 hours talk time to use from your phone
now! ubscribe6GBP
2. mnth inc 3hrs 16 stop?txtStop
3. Sunshine Quiz! Win a super Sony DVD recorder if you canname the capital of Australia? Text MQUIZ
to 82277. B
4. URGENT! Your Mobile No 07808726822 was awarded a L2,000 Bonus Caller Prize on 02/09/03! This is
our 2nd attempt to
contact YOU! Call 0871-872-9758 BOX95QU

**Table 5:** Results on the spam problem. Mean accuracy (Acc.) and area under ROC (AUC) for each of the considered *k* values. The best values are shown in boldface.

*k*	*C*	γ	Acc.	AUC
8	1.5	100	0.8005±0.02	0.85
16	1.5	50	0.827±0.02	0.89
32	1.5	25	0.85±0.01	0.92
80	5	25	0.889±0.02	0.95
160	1.5	25	**0.904** ± **0.01**	**0.96**

**Table 6:** Examples of normal and malicious domains in the DGA dataset.

Normal Domain	Malicious Domain
cfre.org	ofdhiydrrttpblp.com
fabricadericos.com	puciftnfkplcbhp.net
earthrootgaming.com	tahathil.ru
google.com	thisarmedindependent.com
facebook.com	cgoellwcvwti.com
mail.ru	ufaqzt.cn

**Table 7:** Results on the DGA problem. Mean accuracy (Acc.) and area under ROC (AUC) for each of the considered *k* values. The best values are shown in boldface.

*k*	*C*	γ	Acc.	AUC
8	100	25	0.903±0.008	0.96
16	0.5	25	0.918±0.005	0.97
32	0.5	25	0.9305±0.004	0.97
80	1	25	**0.941** ± **0.004**	**0.98**
160	5	25	0.931±0.005	0.97

**Table 8:** A sample of 5 positive and 5 negative tweets extracted from the *Sentiment140 dataset.*

Five Examples of Positive Tweets
1. happy sunny sunday peeps xxx
2. in worrk now chilling out and cleaning the gym oiss easy!! im supervisor today hahahahaha
go me!!!! Clon show!!!!
3. is craving sun chips I think im going to go get some now...lol xo
4. Congratulations! so glad to hear you’ve had a great weekend at the markets
5. I just noticed that I use a lot of smiley faces when I talk. lmfao
**Five Examples of Negative Tweets**
1. I think im going to be pulling a late night to finish this
2. Im sad to see my aunt in jail She was arrested for being a very loud drunk lady
3. grrr bad hayfever day already
4. Furthermore, - I am sunburned. I am hurting Had a good time at meet yesterday, but walked all over
creation and now am very tired.
5. I’m so bored. No one is talking on MSN, there is nothing to do, and I have got no texts..

**Table 9:** Results on the Twitter problem. Mean accuracy (Acc.) and area under ROC (AUC) for each of the considered *k* values. The best values are shown in boldface.

*k*	*C*	Acc.	AUC
8	0.1	0.668±0.003	0.731±0.003
16	10.0	0.699±0.003	0.770±0.003
32	0.01	0.728±0.002	0.805±0.002
80	0.01	0.754±0.002	0.835±0.003
160	0.01	**0.767** ± **0.003**	**0.849** ± **0.002**

**Table 10:** Some examples of positive and negative movie reviews in the Stanford dataset.

Two Examples of Positive Reviews
1. I havent seen that movie in 20 or more years but I remember the attack scene with the horses
wearing gas-masks vividly, this scene ranks way up there with the best of them including the
beach scene on Saving private Ryan, I recommend it strongly.
2. Now this is what I’d call a good horror. With occult/supernatural undertones, this nice
low-budget French movie caught my attention from the very first scene. This proves you do not
need wild FX or lots of gore to make an effective horror movie.
**Two Examples of Negative Reviews**
1. The power rangers is definitely the worst television show and completely ridiculous plastic toy
line in the history of the United States. There is absolutely nothing even remotely
entertaining about this completely awful television show.
2. Some people are saying that this film was "funny". This film is not "funny" at all. Since when
is Freddy Krueger supposed to be "funny"? I would call it funnily crap. This film is supposed
to be a Horror film, not a comedy. If Freddy had a daughter, would not that information have
surfaced like in the first one!? The ending was also just plain stupid and cheesy, exactly
like the rest of it.

**Table 11:** Results on the Movie problem. Mean accuracy (Acc.) and area under ROC (AUC) for each of the considered *k* values. The best values are shown in boldface.

*k*	*C*	γ	Acc.	AUC
8	0.5	0.1	0.6975±0.03	0.78
16	5	0.1	0.68225±0.023	0.75
32	1.5	0.1	0.74925±0.01	0.83
80	20	0.1	0.7227±0.01	0.80
160	0.5	0.1	**0.8590** ± **0.012**	**0.93**

## Data Availability

All the data used in the experiments can be downloaded from the following links: https://www.isi.csic.es/dataset/ (malicious URL, accessed on 13 May 2021), http://www.dt.fee.unicamp.br/~tiago/smsspamcollection/ (spam in SMS messages, accessed on 13 May 2021), https://github.com/andrewaeva/DGA (DGAs, accessed on 13 May 2021), http://help.sentiment140.com (sentiment analysis in Twitter, accessed on 13 May 2021) and http://ai.stanford.edu/~amaas/data/sentiment/ (sentiment analysis in movie reviews, accessed on 13 May 2021).

## Abbreviations

The following abbreviations are used in this manuscript:

ICT	Information and Communication Technologies
APT	Advanced Persistent Threats
DGA	Domain Generation Algorithm
OSINT	Open Source Intelligence
IDS	Intrusion Detection System
NCD	Normalized Compression Distance
SVM	Support Vector Machine
HTTP	Hypertext Transfer Protocol
URL	Uniform Resource Locator
TF	Term Frequency
TFIDF	Term Frequency Inverse Document Frequency
C&C	Command and Control
DNS	Domain Name System
PMI	Point-wise Mutual Information
AG	Attribute Generator
RBF	Radial Basis Function
ACC	Accuracy
AUC	Area Under Curve
SMS	Short Message Service
NLP	Natural Language Processing
IMDb	Internet Movie Database

## References

[1] OECD (2004). The Economic Impact of ICT. https://www.oecd-ilibrary.org/content/publication/9789264026780-en.

[2] Sfakianakis A., Douligeris C., Marinos L., Lourenço M., Raghimi O. (2019). ENISA Threat Landscape Report 2018.

[3] Pastor-Galindo J., Nespoli P., Mármol F.G., Pérez G.M. (2020). The not yet exploited goldmine of OSINT: Opportunities, open challenges and future trends. IEEE Access.

[4] Chuvakin A., Schmidt K., Phillips C. (2013). Logging and Log Management.

[5] Sabottke C., Suciu O., Dumitras T. Vulnerability disclosure in the age of social media: Exploiting twitter for predicting real-world exploits. Proceedings of the 24th USENIX Security Symposium (USENIX Security 15).

[6] Curry S., Kirda E., Schwartz E., Stewart W., Yoran A. (2013). Big data fuels intelligence-driven security. RSA Secur. Brief.

[7] Keogh E., Lonardi S., Ratanamahatana C.A. Towards parameter-free data mining. Proceedings of the Tenth ACM SIGKDD International Conference on Knowledge Discovery and Data Mining.

[8] Ferragina P., Giancarlo R., Greco V., Manzini G., Valiente G. (2007). Compression-based classification of biological sequences and structures via the universal similarity metric: Experimental assessment. BMC Bioinform..

[9] Cilibrasi R., Vitanyi P. Automatic Extraction of Meaning from the Web. Proceedings of the 2006 IEEE International Symposium on Information Theory.

[10] Cilibrasi R., Vitányi P.M.B. (2005). Clustering by compression. IEEE Trans. Inf. Theory.

[11] Yahalom S. (2008). URI Anomaly Detection Using Similarity Metrics. Master’s Thesis.

[12] de la Torre-Abaitua G., Lago-Fernández L.F., Arroyo D. (2017). A parameter-free method for the detection of web attacks. Proceedings of the International Joint Conference SOCO’17-CISIS’17-ICEUTE’17.

[13] Hee C.V., Lefever E., Verhoeven B., Mennes J., Desmet B., Pauw G.D., Daelemans W., Hoste V. Automatic detection and prevention of cyberbullying. Proceedings of the International Conference on Human and Social Analytics (HUSO 2015).

[14] Killam R., Cook P., Stakhanova N. Android malware classification through analysis of string literals. Proceedings of the First Workshop on Text Analytics for Cybersecurity and Online Safety (TA-COS).

[15] Hernandez-Suarez A., Sanchez-Perez G., Toscano-Medina K., Martinez-Hernandez V., Meana HM P., Olivares-Mercado J., Sanchez V. (2018). Social sentiment sensor in twitter for predicting cyber-attacks using L1 regularization. Sensors.

[16] García-Teodoro P., Díaz-Verdejo J., Maciá-Fernández G., Vázquez E. (2009). Anomaly-based network intrusion detection: Techniques, systems and challenges. Comput. Secur..

[17] Bhuyan M.H., Bhattacharyya D.K., Kalita J.K. (2014). Network Anomaly Detection: Methods, Systems and Tools. IEEE Commun. Surv. Tutorials.

[18] Chaurasia M.A. (2016). Comparative study of data mining techniques in intrusion dectection. Int. J. Curr. Eng. Sci. Res..

[19] Dong Y., Zhang Y. (2017). Adaptively Detecting Malicious Queries in Web Attacks. arXiv.

[20] Hodo E., Bellekens X., Hamilton A., Tachtatzis C., Robert A. (2017). Shallow and Deep Networks Intrusion Detection System: A Taxonomy and Survey. arXiv.

[21] Kruegel C., Vigna G. Anomaly Detection of Web-based Attacks. Proceedings of the 10th ACM Conference on Computer and Communications Security.

[22] Abu-Nimeh S., Nappa D., Wang X., Nair S. A comparison of machine learning techniques for phishing detection. Proceedings of the Anti-phishing Working Groups 2nd Annual eCrime Researchers Summit, eCrime ’07.

[23] Mallikarjunappa B., Prabhakar D.R. (2010). A novel method of spam mail detection using text based clustering approach. Int. J. Comput. Appl..

[24] Tee H. (2010). FPGA Unsolicited Commercial Email Inline Filter Design Using Levenshtein Distance Algorithm and Longest Common Subsequence Algorithm.

[25] Delany S.J., Bridge D., Weber R.O., Richter M.M. (2007). Catching the drift: Using feature-free case-based reasoning for spam filtering. Case-Based Reasoning Research and Development.

[26] Prilepok M., Berek P., Platos J., Snasel V. (2013). Spam detection using data compression and signatures. Cybern. Syst..

[27] Bratko A., Filipič B., Cormack G.V., Lynam T.R., Zupan B. (2006). Spam filtering using statistical data compression models. J. Mach. Learn. Res..

[28] Antonakakis M., Perdisci R., Nadji Y., Vasiloglou N., Abu-Nimeh S., Lee W., Dagon D. From throw-away traffic to bots: Detecting the rise of dga-based malware. Proceedings of the 21st USENIX Conference on Security Symposium, Security’12.

[29] Thomas M., Mohaisen A. Kindred domains: Detecting and clustering botnet domains using dns traffic. Proceedings of the 23rd International Conference on World Wide WebWWW ’14 Companion.

[30] Ahluwalia A., Traore I., Ganame K., Agarwal N., Traore I., Woungang I., Awad A. (2017). Detecting broad length algorithmically generated domains. Intelligent, Secure, and Dependable Systems in Distributed and Cloud Environments.

[31] Woodbridge J., Anderson H.S., Ahuja A., Grant D. (2016). Predicting domain generation algorithms with long short-term memory networks. arXiv.

[32] Selvi J., Rodríguez R.J., Soria-Olivas E. (2019). Detection of algorithmically generated malicious domain names using masked n-grams. Expert Syst. Appl..

[33] Tong V., Nguyen G. A method for detecting dga botnet based on semantic and cluster analysis. Proceedings of the Seventh Symposium on Information and Communication Technology, SoICT ’16.

[34] Aslan Ç.B., Li S., Çelebi F.V., Tian H. (2020). The World of Defacers: Looking through the Lens of Their Activities on Twitter. IEEE Access.

[35] Al-Rowaily K., Abulaish M., Al-Hasan Haldar N., Al-Rubaian M. (2015). Bisal—A bilingual sentiment analysis lexicon to analyze dark web forums for cyber security. Digit. Investig..

[36] Weifeng L., Hsinchun C. Identifying top sellers in underground economy using deep learning-based sentiment analysis. Proceedings of the 2014 IEEE Joint Intelligence and Security Informatics Conference.

[37] Zaeem R.N., Li C., Barber K.S. On Sentiment of Online Fake News. Proceedings of the 2020 IEEE/ACM International Conference on Advances in Social Networks Analysis and Mining (ASONAM).

[38] Zollo F., Novak P.K., Del Vicario M., Bessi A., Mozetič I., Scala A., Caldarelli G., Quattrociocchi W. (2015). Emotional dynamics in the age of misinformation. PLoS ONE.

[39] Deb A., Lerman K., Ferrara E. (2018). Predicting cyber events by leveraging hacker sentiment. Information.

[40] Mittal S., Das P.K., Mulwad V., Joshi A., Finin T. Cybertwitter: Using twitter to generate alerts for cybersecurity threats and vulnerabilities. Proceedings of the 2016 IEEE/ACM International Conference on Advances in Social Networks Analysis and Mining (ASONAM).

[41] Liu B., Zhang L. (2012). A survey of opinion mining and sentiment analysis. Mining Text Data.

[42] Medhat W., Hassan A., Korashy H. (2014). Sentiment analysis algorithms and applications: A survey. Ain Shams Eng. J..

[43] dos Santos C., Gatti M. Deep convolutional neural networks for sentiment analysis of short texts. Proceedings of the 25th International Conference on Computational Linguistics, COLING 2014.

[44] Severyn A., Moschitti A. Twitter sentiment analysis with deep convolutional neural networks. Proceedings of the 38th International ACM SIGIR Conference on Research and Development in Information Retrieval.

[45] Tang D., Wei F., Qin B., Liu T., Zhou M. Coooolll: A deep learning system for twitter sentiment classification. Proceedings of the 8th International Workshop on Semantic Evaluation (SemEval 2014).

[46] (2005). Tukaani-Project. A Quick Benchmark: Gzip vs. Bzip2 vs. LZMA. http://tukaani.org/lzma/benchmarks.html.

[47] Scikit Learn Scikit-Learn: Machine Learning in Python—Scikit-Learn 0.18.1 Documentation. http://scikit-learn.org/stable/.

[48] Torrano-Gimenez C., Nguyen H.T., Alvarez G., Franke K. (2015). Combining expert knowledge with automatic feature extraction for reliable web attack detection. Secur. Commun. Netw..

[49] CSIC-Dataset (2010). HTTP DATASET CSIC. http://www.isi.csic.es/dataset/.

[50] Nguyen H.T., Torrano-Gimenez C., Alvarez G., Petrović S., Franke K. (2011). Application of the generic feature selection measure in detection of web attacks. Computational Intelligence in Security for Information Systems.

[51] Manning C., Surdeanu M., Bauer J., Finkel J., Bethard S., McClosky D. The stanford corenlp natural language processing toolkit. Proceedings of the 52nd Annual Meeting of the Association for Computational Linguistics: System Demonstrations.

[52] Almeida T.A., Hidalgo J.M.G., Yamakami A. Contributions to the study of sms spam filtering: New collection and results. Proceedings of the 11th ACM Symposium on Document Engineering DocEng ’11.

[53] Lison P., Mavroeidis V. (2017). Automatic detection of malware-generated domains with recurrent neural models. arXiv.

[54] Go A., Bhayani R., Huang L. (2009). Twitter Sentiment Classification Using Distant Supervision.

[55] Maas A.L., Daly R.E., Pham P.T., Huang D., Ng A.Y., Potts C. (2011). Learning word vectors for sentiment analysis. Proceedings of the 49th Annual Meeting of the Association for Computational Linguistics: Human Language Technologies.

[56] Samtani S., Kantarcioglu M., Chen H. (2020). Trailblazing the Artificial Intelligence for Cybersecurity Discipline: A Multi-Disciplinary Research Roadmap. ACM Trans. Manag. Inf. Syst..

[57] Lillis D., Becker B., O’Sullivan T., Scanlon M. (2016). Current challenges and future research areas for digital forensic investigation. arXiv.

[58] de la Torre-Abaitua G., Lago-Fernández L., Arroyo D. (2020). On the application of compression based metrics to identifying anomalous behaviour in web traffic. Log. J. IGPL.

[59] Resende J.S., Martins R., Antunes L. (2019). A Survey on Using Kolmogorov Complexity in Cybersecurity. Entropy.

[60] Larriva-Novo X., Sánchez-Zas C., Villagrá V.A., Vega-Barbas M., Rivera D. (2020). An Approach for the Application of a Dynamic Multi-Class Classifier for Network Intrusion Detection Systems. Electronics.

[61] Wolpert D.H., Macready W.G. (1997). No free lunch theorems for optimization. IEEE Trans. Evol. Comput..

